# Effect of Calcium Chloride as a Coagulant on the Properties of ESBR/Silica Wet Masterbatch Compound

**DOI:** 10.3390/polym10101116

**Published:** 2018-10-09

**Authors:** Woong Kim, Byungkyu Ahn, Hyunsung Mun, Eunho Yu, Kiwon Hwang, Donghyuk Kim, Gyeongchan Ryu, Wonho Kim

**Affiliations:** Department of Polymer Science & Chemical Engineering, Pusan National University, Busandaehak-ro 63beon-gil, Geumjeong-gu, Busan 46241, Korea; kw65651294@gmail.com (W.K.); bkahn8855@gmail.com (B.A.); ansehdwns10@gmail.com (H.M.); yueh5352@gmail.com (E.Y.); kiwon8348@gmail.com (K.H.); ehdgurzxc@gmail.com (D.K.); 60chan95@gmail.com (G.R.)

**Keywords:** polymer composite, silica filled compound, rubber, emulsion styrene-butadiene rubber/silica wet masterbatch, silica dispersion

## Abstract

When designing rubber compounds for high-performance tires, increasing the silica content can improve the wet traction performance but decreases the fuel efficiency. This trade-off relation makes it difficult to improve the two factors simultaneously. One approach is the development of silica wet masterbatch (WMB) technology for producing compounds containing a high silica content with good dispersion. The technology involves a step to mix surface-modified silica and rubber latex. The technique requires a coagulant to break up the micelles of the rubber latex and cause the surface-modified silica and the rubber molecules to co-coagulate due to van der Waals forces. In this study, the effect of coagulant type on the characteristics of silica surface, and the mechanical properties of the emulsion styrene-butadiene rubber (ESBR)/silica WMB compounds was investigated, as well as the abrasion properties and the viscoelastic properties of the vulcanizates.

## 1. Introduction

Rolling resistance and wet traction are the important factors in tire performances related to fuel consumption and safety during the driving of the vehicle. These factors must be considered in the design of rubber compounds used for tire tread [1,2,3,4]. In general, it is known that the good wet traction performance of a tire (high tan δ value at 0 °C) is shown when there is high filler content, functionalized polymer with high filler-rubber affinity, and good filler dispersion [4,5,6]. The rolling resistance is also known to be lower (good fuel efficiency characteristics) when tan δ at 60 °C of the vulcanizates is lowered by the time-temperature superposition principle. Highly effective methods for low rolling resistance include reducing the filler content, improving filler dispersion, and using functionalized polymer with high filler affinity [3,6,7]. In other words, improving both the fuel efficiency and wet traction performance of the tire requires the use of functionalized polymers and high content as well as best dispersion of reinforcing agent in the tread rubber compound [6,8]. However, when functionalized polymer is used, the raw material cost is higher than that of non-functionalized polymer [9], so increasing the content and improving filler dispersion are the priority.

Currently, silica is the dominant filler used in the tire industry. Hydrophilic silanol groups (Si–OH) are present on the silica surface, so the interaction with hydrophobic unfunctionalized polymer is low and it is difficult to obtain sufficient filler-rubber interaction (F–R interaction), even when silica-friendly functional polymer is used. Therefore, a silane coupling agent must be used to effectively disperse silica in silica filled rubber compounds by silanization reaction [10,11]. However, despite the use of a silane coupling agent, the filler-filler interaction (F–F interaction) is still strong due to hydrogen bonding between the residual silanol groups when a high content of silica is added, which remains a disadvantage [12]. F–F interactions are particularly problematic when high silica content in tire tread compounds is used to improve wet traction performance. The result is that the rolling resistance is increased when silica content is increased [6]. Therefore, in order to simultaneously improve both performances in the trade-off relationship, a new material is required, containing a high content of silica with a good dispersion. To prepare this material, silica wet masterbatch (silica WMB) technology has been developed [13,14].

Silica WMB technology refers to a technique of preparing composites of rubber/silica/silane coupling agent by mixing a rubber solution or rubber latex with surface-modified silica, followed by a stripping or coagulation process. The advantages of this technology are the production of silica compounds with a high silica content and good dispersion, improved energy efficiency of the tire manufacturing process, reduced emission of ethanol, and lower hysteresis (lower rolling resistance) [15,16,17].

Currently, silica WMB technology researchers at petrochemical and tire companies are mainly focused on manufacturing and evaluating the solution styrene-butadiene rubber (SSBR)/silica WMB. These studies showed that SSBR/silica WMB compounds had a 20% lower rolling resistance and 18% improved wet traction performance than the DMB compound (dry masterbatch, conventional mixing). However, SSBR/silica WMB technology had a problem, as it generated organic solvent vapor during stripping [18,19]. In the university laboratory scale, it is difficult to treat and recover the organic solvent vapor generated during the stripping process. Therefore, emulsion styrene-butadiene rubber (ESBR)/silica WMB and natural rubber (NR)/silica WMB using rubber latex are mainly studied, which use the coagulation technique to obtain WMB compounds [20,21,22].

**Figure 1:**
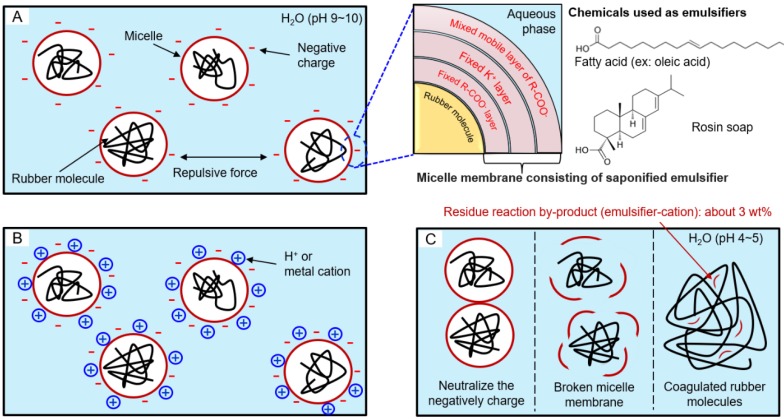
Coagulation mechanism of synthetic rubber such as emulsion styrene-butadiene rubber (ESBR): (**A**) a state in which a micelle having a negative surface charge is dispersed in water, (**B**) a state after adding coagulant in latex, and (**C**) a state of micelle destruction due to collision between neutralized micelles.

In general, micelles dispersed in water by a saponified emulsifier surrounding a rubber molecule, are called a rubber latex. The latex shows basicity (pH 9~10) due to the OH anion from KOH used in the saponification of emulsifier. By the addition of coagulant into the latex, coagulation will be started [23,24,25]. Coagulation mechanism is shown in Figure 1.

However, the detailed reaction mechanism (reactions between the emulsifier and the coagulant and between the coagulant and silica) that occurs during the preparation of emulsion silica WMB is not yet known. Also, there are insufficient researches on the compounds which applied application of WMB technology. However, we studied about the best blending method of ESBR WMB/butadiene rubber (BR) in our previous study [26]. In this study, we researched the effect of coagulants on the properties of ESBR/silica compounds. In detail, we determined the reaction mechanism of emulsion SBR silica WMB and the suitable manufacturing conditions. The study was carried out using three types of coagulants commonly used in manufacturing of ESBR/silica WMB; aqueous solutions of 1 M sulfuric acid, 25 wt % sodium chloride, and 2 wt % calcium chloride. The effect of coagulants on the silica dispersion, mechanical properties, and dynamic viscoelastic properties of ESBR/silica WMB compounds was investigated. We also studied specific improvements of ESBR/silica WMB by comparing the properties with those of existing DMB compound.

## 2. Materials and Experimental Methods

### 2.1. Materials

To determine the optimal type of modified silica for ESBR/silica WMB, the degree of hydrophobation of silica (Newsil 175, Quechen Silicon Chemical CO., Ltd., Wuxi, China, BET surface area: 175 m^2^/g) treated with 8, 10, 12, 15 wt % of bis[3-(triethoxysilyl)propyl]tetrasulfide (TESPT, Evonik, Essen, Germany) to silica was compared. Detailed information of TESPT-modified silica was shown in Table 1. 

After these experiments (Section 2.2), the materials prepared using 12 wt % were selected for further use in ESBR/silica WMB experiments. The ESBR latex and processing oil used were SBR-1712 (Kumho Petrochemical Co., Daejeon, South Korea, styrene content: 23.5%, non-oil extended) and treated distillate aromatic extracted (TDAE) oil. For the coagulation process of ESBR/silica WMB, the coagulants used were aqueous solutions of 1 M sulfuric acid (Daejung Chemical & Metals Co. Siheung, South Korea), 25 wt % sodium chloride (Samchun Chemical, Seoul, South Korea), and 2 wt % calcium chloride (Samchun Chemical).

The DMB compound (T-1) was prepared for comparison with the WMB compounds using pure silica, TESPT, and SBR-1723 (Kumho Petrochemical Co., styrene content: 23.5 wt %, 37.5 phr TDAE oil extend). Zinc oxide (ZnO) and stearic acid (St/A), which act as activators, and *N*-(1,3-dimethyl-butyl)-*N*′-phenyl-p-phenylenediamine (6PPD) as an antioxidant were added during the compounding process of the kneader. In the final masterbatch (FMB) step, sulfur, *n*-cyclohexyl-2-benzothiazole sulfonamide (CBS), and diphenyl guanidine (DPG) were added as crosslinking agents and cure accelerators.

To analyze the effect of the alkali-silica reaction, which is the reaction between the calcium cation and the silanol group on the silica surface, Ca-coated 12 wt % TESPT-modified silica was manufactured using calcium hydroxide (Daejung Chemical & Metals Co.) and ethanol (Daejung Chemical & Metals Co.).

The hydrophobation of modified silica was measured using di-butylamine (>99.5%, Sigma Aldrich, St. Louis, MI, USA), petroleum benzine (>90%, Samchun Chemical), chloroform (>99.5%, Samchun Chemical), crystal violet indicator (>90%, Sigma Aldrich), perchloric acid (>70%, Samchun Chemical), and acetic anhydride (>99%, Samchun Chemical). The crosslink density was measured using tetrahydrofuran (>99.5%, Daejung Chemical & Metals Co.), *n*-hexane (>96%, Daejung Chemical & Metals Co.), toluene (>99.5%, Daejung Chemical & Metals Co.), and acetone (>99.9%, Daejung Chemical & Metals Co.).

### 2.2. Experimental Methods

#### 2.2.1. Analysis of Modified Silica and Ca-Coated TESPT-Modified Silica

Di-*n*-butylamine (DBA) adsorption analysis can quantitatively demonstrate the hydrophobation of surface-modified silica. The degree of hydrophobation of modified silica can be measured by titrating the amount of unabsorbed DBA to the silica silanol groups.

In detail, after the surface-modified silica was dried at 105 °C for 2 h, 250 mg of the obtained silica was added to 50 mL of petroleum benzine solution in which 0.002 N DBA was dissolved and then stored at 20 °C for 2 h. Then, 25 mL of the upper layer solution was sampled, mixed with 5 mL of chloroform and 2–3 drops of crystal violet indicator, and then titrated with acetic anhydride solution containing 0.01 N perchloric acid. When the color of the solution changed from violet to blue, the volume (mL) of the perchloric acid/acetic anhydride solution added up to this point was determined [27]. We measured the titration solution volume value of each sample 3 times and then obtained an average value (mL).

Substituting the calculated volume of titration solution into Equation (1) gives the number of moles of DBA adsorbed on the silica surface, which can then be substituted into Equation (2) to obtain the hydrophobation of the modified silica.

(1) DBA adsorption (mmolkg)=80∗(β−α)∗f

where *α* is the titration value (mL) when color change occurs with silica, *β* is the titration value (mL) when color change occurs without silica, and *f* is the chemical potency of 0.01 N perchloric acid solution.

(2) Hydrophobation ratio(%) = 100−(DBA/DBA’)×100

where DBA (mmol·kg^−1^) is the DBA adsorption on modified silica and DBA’ (mmol·kg^−1^) is the DBA adsorption on pure silica.

To analyze the effect of the alkali-silica reaction, which is a reaction between the calcium ions produced by ionization from calcium chloride used as a coagulant and the silanol groups of silica, Ca-coated TESPT-modified silica was manufactured using similar conditions to those used for ESBR/silica WMB manufacturing [28]. First, surface-modified silica slurry was prepared by mixing 12 wt % TESPT-modified silica with distilled water for 15 min. In order to show the same pH value of calcium ion supply and basic rubber latex, 25 wt % calcium hydroxide aqueous solution was added to the above surface-modified silica slurry to adjust the pH value to 9. After that, the Ca-coated 12 wt % TESPT-modified silica was filtered from the silica slurry using a vacuum filtration apparatus and washed 3 times with ethanol. The prepared Ca-coated TESPT-modified silica was analyzed using X-ray fluorescence (EDX-7000, Shimadzu, Kyoto, Japan) and analyzed for hydrophobation by DBA adsorption.

#### 2.2.2. Manufacturing of ESBR/Silica WMBs

The 89.6 phr of 12 wt % TESPT-modified silica was added to 1 L distilled water and the mixture was stirred at 60 °C for 15 min to prepare a modified silica slurry. The modified silica slurry was mixed with SBR-1712 latex containing 100 phr solid ESBR, heated to 60 °C and further stirred for 30 min. And added 37.5 phr TDAE oil emulsion into heated mixture, stirring for 30 min. WMBs were prepared using three types of coagulants: 1 M aqueous sulfuric acid solution 200 mL (WMB T-2), 1 M aqueous sulfuric acid solution 15 mL combined with a 25 wt % sodium chloride solution 300 mL (WMB T-3), and 2 wt % calcium chloride aqueous solution 300 mL (WMB T-4). Thereafter, ESBR/silica WMBs were washed 4 times using distilled water before drying in a convection oven at 60 °C for 12 h. 

#### 2.2.3. Characterization of WMBs

The silica content of ESBR/silica WMB was measured by thermogravimetric analysis (TGA 550, TA Instruments Korea, Seoul, South Korea). The measurement was carried out by increasing the temperature from room temperature to 900 °C at a rate of 10 °C·min^−1^ under a nitrogen atmosphere, and then measuring the silica content by substituting the measured data into Equation (3).

(3) $$\mathrm{Silica}\;\left(\mathrm{phr}\right)=\frac{A}{B}\times 100$$

where *A* is the ash percentage (pure silica weight percent) and *B* is the weight loss percentage between 360 and 900 °C (SBR weight percent).

In addition, the acidity (pH) of ESBR/silica WMB was indirectly measured in order to investigate additional properties depending on the type of coagulant. For the detailed procedure, 1 g of dried ESBR/silica WMB sample was immersed in 50 mL of distilled water. After 2 h of storage at 50 °C, the WMB sample was filtered and the pH of the residual solution was measured 3 times using a pH meter [29].

#### 2.2.4. Compounding of WMB Compounds and DMB Compound

The rubber compound was prepared by applying a fill factor of 0.7 using a closed mixer (300 cc, Miraesi Inc.). Compounding was carried out in a silica masterbatch (SMB) stage at a starting temperature of 120 °C for 12 min, dump temperature was 155 °C. The detailed formulations and mixing procedures for T-1 to T-4 are shown in Table 2 and Table 3.

#### 2.2.5. Analysis of Uncured Compounds

The bound rubber content was measured to determine the degree of F–R interaction, we prepared 4 samples of each per 1 compound then obtained average bound rubber contents (%) by bound rubber analysis. In detail, bound rubber content was measured by adding 0.2 g of unvulcanized rubber compound to 200 mesh wire net and storing in a vial containing 200 mL of toluene for six days. The toluene was replaced once on the third day. After that, the solvent was replaced with acetone to remove the toluene and stored for 24 h. Then, the mass was measured after drying in an oven at 10.5 °C for 24 h. The measured weight was substituted into Equation (4) to calculate the bound rubber content.

(4) $$R_{b}\;\left(\%\right)=\frac{W_{\mathrm{fg}}-W_{t}\left[\frac{m_{f}}{m_{f}+m_{r}}\right]}{W_{t}\left[\frac{m_{r}}{m_{f}+m_{r}}\right]}*100$$

where *R*_b_ is the bound rubber content (%), *W*_fg_ is the weight of the filler and gel, *W*_t_ is the weight of the sample before immersion, m_f_ is the weight fraction of the filler in the compound, and *m*_r_ is the weight fraction of rubber in the compound.

The Moony viscosity was measured 2 times according to ASTM D 1646 using a Moony viscometer (Vluchem IND, Siheung, Korea). In detail, samples were preheated for 1 min at 100 °C and then rotated for 4 min at 2 RPM at 100 °C. The Payne effect, which is an index of silica dispersibility, was measured according to ASTM D 8059 using a rubber process analyzer (RPA 2000, Alpha Technologies, Hudson, OH, USA), based on the results of a study by the Dutch Society of Plastic and Rubber Technologists [30]. The Payne effect was calculated using the *G*′ value at a strain interval of 1.8–31.7%.

#### 2.2.6. Analysis of WMB and DMB Vulcanizates

The sheeted FMB compound prepared by a two-roll mill, which was tested to analyze the cure characteristics using a moving die rheometer (MDR, TOYOSEIKI, Tokyo, Japan) for 30 min at 160 °C with vibration conditions of ±1°. The cure rate was calculated using the measured values, and the vulcanization was carried out at 160 °C for the measured cure characteristics. 

To measure the crosslinking density of the vulcanized compounds, they were cut into dimensions of 10 mm × 10 mm and stored in a vial containing tetrahydrofuran (THF, 50 mL) for 48 h. The sample was then treated with n-hexane (50 mL) for 48 h to remove organic chemicals. After drying for two days at room temperature, the mass of the sample was measured, and then the weight was measured by swelling in toluene for 48 h. The measured weight values were substituted into Equation (5) to calculate the crosslink density.

(5) $$\upsilon =\frac{1}{2M_{c}}=-\frac{\ln\left(1-V_{1}\right)+V_{1}+\chi {V_{1}}^{2}}{2\rho_{r}V_{0}\left({V_{1}}^{\frac{1}{3}}-\frac{V_{1}}{2}\right)}$$

where *ν* is the crosslink density (mol·g^−1^), *M*_c_ is the average molecular weight between crosslink points (g·mol^−1^), *V*_1_ is the volume fraction of rubber in the swollen gel at equilibrium, *V*_0_ is the molar volume of solvent (cm^3^·mol^−1^), *ρ*_r_ is the density of the rubber sample (g·cm^−3^), and *χ* is the polymer-solvent interaction parameter.

To measure the mechanical properties of the vulcanizates, 3 specimens per each compound were prepared according to ASTM D 412 and evaluated using a universal testing machine (UTM, KSU Co., Seoul, South Korea). The abrasion resistance was evaluated with a DIN abrasion tester (KSU Co., Korea) according to ASTM D 5963. Four specimens per each compound were manufactured in a cylindrical shape with a diameter of 16 mm and a thickness of 8 mm and the mass change of the specimens was measured by abrading the specimen with 40 m under a vertical load of 5 N using a cylindrical drum with a polishing cloth. 

The dynamic viscoelastic properties of the vulcanizates were measured according to ASTM D 4065 using a dynamic mechanical thermal analyzer (EPLEXOR 500 N, GABO, Ahlden, Germany) under the temperature range of −80 to 80 °C. The glass transition temperature (*T*_g_), tan δ at 0 °C (wet traction index), and tan δ at 60 °C (rolling resistance index) were measured. 

## 3. Results and Discussion

### 3.1. Hydrophobation of TESPT-Modified Silica

Table 4 shows the results of hydrophobation of surface-modified silica according to TESPT content. As the TESPT content increased, the hydrophobation of silica also increased, but the reaction efficiency decreased. This is due to the steric hindrance caused by the previously reacted TESPT molecules when the incoming TESPT reacts with the silanol groups on the silica surface [31]. Therefore, in this study, 12% TESPT-modified silica was used in subsequent experiments, because 10% TESPT silica showed lower hydrophobation than 12% TESPT silica. Also, 15% TESPT silica showed higher hydrophobation than 12% TESPT silica, but it had shown low efficiency for hydrophobation.

### 3.2. Silica Content and pH of ESBR/Silica WMBs

**Figure 2:**
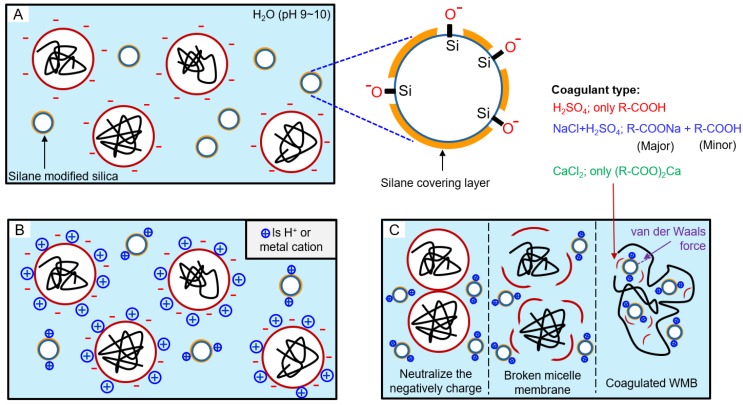
Illustration of the reaction among surface-modified silica, surfactant, and coagulant during WMB manufacturing process: (**A**) a state in which micelles and modified silica having a negative charge surface are dispersed in water, (**B**) a state after coagulants were added, and (**C**) a state in which modified silica and ESBR were coagulated. Also, by-products between emulsifier and cation remained, for example, carboxy acid, sodium carboxylate, and calcium carboxylate, according to the coagulant type.

The pH values of ESBR/silica WMB prepared using different types of coagulants were indirectly measured and the results are shown in Table 5. As a result, ESBR/silica WMB with sulfuric acid solution alone showed the lowest pH value, and WMB with the mixture of sodium chloride solution and sulfuric acid solution was lower than 7. This is the result of the pH being lowered by residual sulfuric acid and the fatty acid (R–COOH), the reaction product of the surfactant and coagulant shown in Figure 2. WMB T-4, which used the calcium chloride solution, showed a pH value higher than 7 since a compound of calcium carboxylate ((R–COO)_2_Ca) is formed without generating a fatty acid type compound when a coagulant and an emulsifier are reacted.

The silica content of ESBR/silica WMB was analyzed by TGA, and the results are shown in Table 6 and Figure S1. As a result of the evaluation, WMB T-4, which used a calcium chloride aqueous solution containing a divalent metal atom as a coagulant, showed the highest silica content. When ESBR/silica WMB is manufactured, surface-modified silica and ESBR were coagulated due to van der Waals force [32]. Therefore, the higher degree of silica hydrophobation cause the higher silica content of ESBR/silica WMB. 

When the calcium chloride solution is applied as a coagulant in the basic condition, the hydrogen of the unreacted silanol group on the silica surface is removed in basic condition, then calcium cation reacted with SiO^−^ on the silica surface. As a result, the silica surface is more hydrophobized by the alkali-silica reaction and the reaction between the emulsifier and coagulant, as shown in Figure 4 [29,33]. The second highest result was obtained in the WMB T-3. Since the Na^+^ ion is a monovalent cation, thus has lower reactivity with the silanol group than the Ca^2+^ ion [33,34]. 

When sulfuric acid was applied alone, the alkali-silica reaction could not take place, and the silanol group remained as it was, thus showing the lowest silica content. 

### 3.3. Analysis of Uncured Compounds

**Figure 3:**
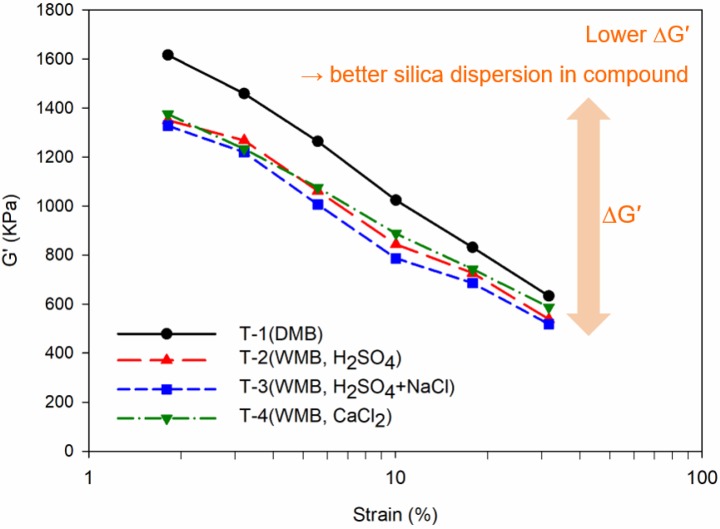
Payne effect (Δ*G*′) of the T-1 to T-4 compounds, which mean degree of filler dispersion in a compound: the black line graph is the Payne effect result of the T-1 DMB compound, the red line is the Payne effect result of the T-2 WMB compound, the blue line is the Payne effect result of the T-3 WMB compound, and the green line is the Payne effect result of the T-4 WMB compound.

**Figure 4:**
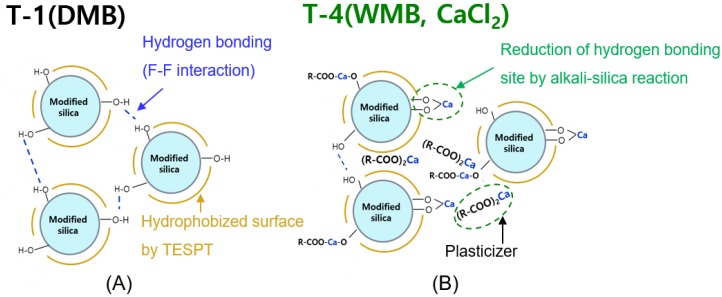
Illustration of expected filler network structure in the silica filled compounds: (**A**) the T-1 (DMB) compound, which has a residue silanol group and interaction between silica by hydrogen bonding and (**B**) The T-4 WMB compound using CaCl_2_, which has fewer silanol groups than the DMB compound by alkali-silica reaction.

Figure 3 and Table 7 show the results of the Mooney viscosity (ML_1+4_@100 °C) and Payne effect (Δ*G*′, *G*_initial_ − *G*_final_) analysis, which is a measure of processability and silica dispersion in compounds. Generally, low ML_1+4_@100 °C value and low Δ*G*′ value show good processability because of the good silica dispersion [30,35,36]. The ESBR/silica WMB compound showed lower Mooney viscosity than the DMB compound. As shown in Figure 4, Mooney viscosity results caused by the lubricant role of the aforementioned metal carboxylate compounds and the increased hydrophobation of the silica due to the alkali-silica reaction. 

The results of the Payne effect also show that the ESBR/silica WMB compound exhibits lower Δ*G*′ value than DMB compound. In particular, the lowest Δ*G*′ value occurs when calcium chloride is added due to the increased hydrophobation of the silica via the alkali-silica reaction. Combining the results of the two analyses, WMB compounds showed good silica dispersion; in particular, the WMB compound showed the best silica dispersion in the compound using calcium chloride as a coagulant.

Bound rubber measurement results are shown in Table 8. The T-4 compound with calcium chloride as a coagulant showed high bound rubber content and showed excellent results with respect to the F–R interaction. The F–R interaction is improved as the hydrophobation of the silica is increased by the alkali–silica reaction. However, T-2 and T-3 compounds containing sulfuric acid showed lower bound rubber content than the DMB compound, which was disadvantageous in the F–R interaction.

### 3.4. Characterization of Ca-Coated TESPT-Modified Silica

Table 9 shows the hydrophobation results of Ca-coated 12 wt % TESPT-modified silica by DBA adsorption and the calcium content analysis by XRF. The Ca-coated 12 wt% TESPT-modified silica showed a 10% increase in hydrophobation compared to the conventional 12 wt% modified silica, and the calcium content of the silica increased by 14 times after the actual calcium treatment. These results showed by alkali-silica reaction between remaining silanol groups on the surface of TESPT modified silica and Ca cation from calcium hydroxide. Thus, the concentration of hydrogen atom decreased on the Ca-coated modified silica surface, and active sites for hydrogen bonding reduced. It caused a higher hydrophobation value than that of un-coated TESPT modified silica. Therefore, the alkali-silica reaction between silanol groups and calcium ions will occur under similar conditions in ESBR/silica WMB using calcium chloride as a coagulant, which results in excellent silica dispersion in the ESBR/silica WMB compound. 

### 3.5. Cure Characteristics and Crosslink Density

**Figure 5:**
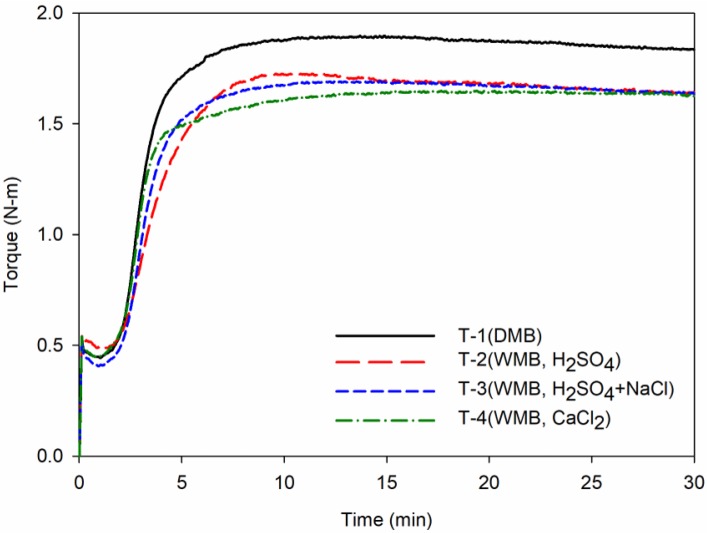
Cure characteristics of the T-1 to T-4 compounds: Each graph shows cure characteristics behavior of compounds according to the cure time. The black line shows the cure behavior of the T-1 DMB compound, the red line shows the T-2 WMB compound, the blue line shows the T-3 WMB compound, and the green line shows the T-4 WMB compound.

The results of the measurement of the cure characteristics and the crosslink density of the vulcanizates are shown in Figure 5 and Table 10. As a result, the WMB compound showed a lower *T*_max_ value and delta torque (*T*_max_ − *T*_min_) value than the DMB compound. It was found that the metal carboxylate or fatty acid compound formed by the reaction between coagulant and surfactant acted as a lubricant [37]. In the case of the T-2 and T-3 compounds using sulfuric acid as a coagulant, the activity of the accelerator declined due to the acid-base reaction of residual sulfuric acid and accelerator, resulting in a slow cure rate compared with that of the DMB compound. However, when calcium chloride was added, the acid-base reaction does not occur with the accelerator because there is no residual acid. Adsorption loss due to the silanol group was also low as the hydrophobation further increased through the alkali-silica reaction, indicating that the cure rate was faster than that of the DMB compound.

As a result of the crosslink density measurement, the crosslink density values of the WMB compounds were lower than that of the DMB compound. This is because the crosslink density of the ESBR WMB compound using a sulfuric acid coagulant was decreased by residual sulfuric acid, and that of the WMB compound using calcium chloride was decreased by the metal carboxylate compound. According to the results of the study of calcium carboxylate added to rubber compounds [37,38], calcium stearate added at 0.5 phr improved the dispersion and mechanical properties of silica. However, the addition of more than 0.5 phr of calcium stearate inhibited the reaction of ZnO, resulting in decreased crosslink density. Therefore, in this ESBR/silica WMB manufacturing process, the surfactant reacts with calcium ions to produce a calcium carboxylate compound in the form of a similar calcium stearate. Therefore, it is considered that the produced calcium carboxylate compound is present in the rubber compound at 0.5 phr or more. 

### 3.6. Mechanical Properties and DIN Abrasion of T-1 to T-4 Vulcanizates

**Figure 6:**
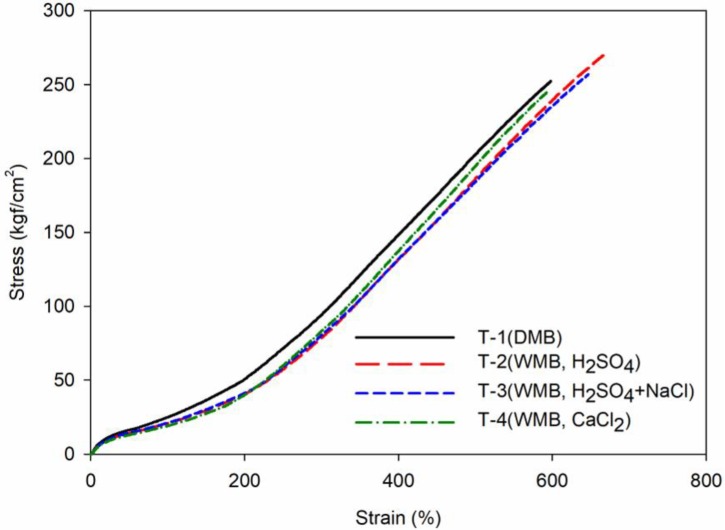
Mechanical properties of the T-1 to T-4 compounds: the black line shows the mechanical property of the DMB T-1 compound, the red line shows the mechanical property of the WMB T-2 compound, the blue line shows the mechanical property of the WMB T-3 compound, and the green line shows the mechanical property of the WMB T-4 compound.

The mechanical properties are shown in Figure 6 and Table 11. The 100% modulus exhibits a higher value when the silica dispersion is poor. Therefore, when comparing the T-3 and T-4 compounds, the T-4 compound shows a low 100% modulus, confirming the excellent silica dispersion. The T-1 compound with the highest crosslink density showed the highest 300% modulus. The T-3 and T-4 compounds showed similar crosslinking densities, but as the T-4 compound had excellent F–R interaction, the 300% modulus for the T-4 compound was better. The T-2 compound showed the most unfavorable value due to the low crosslink density and F–R interaction. In the mechanical properties test, the value obtained by dividing the 300% modulus by the 100% modulus is called a reinforcement effect and is used as a measure of F–R interaction on mechanical properties. The results show that the T-4 compound has the highest value, which is consistent with the results of the bound rubber content evaluation. Tensile strength and elongation were found to be similar for all compounds. 

The DIN abrasion test showed that the T-4 compound had the best value to support the bound rubber test result and the reinforcing effect calculation result. The DMB compound, T-1, also showed good results. The T-2 and T-3 compounds have a more adverse result than T-1 compounds in the same way as the outcome trends in the bound rubber content, in particular, T-2, a compound using sulfuric acid alone, had the least favorable results due to its low crosslink density.

### 3.7. Viscoelastic Properties of T-1 to T-4 Vulcanizates

**Figure 7:**
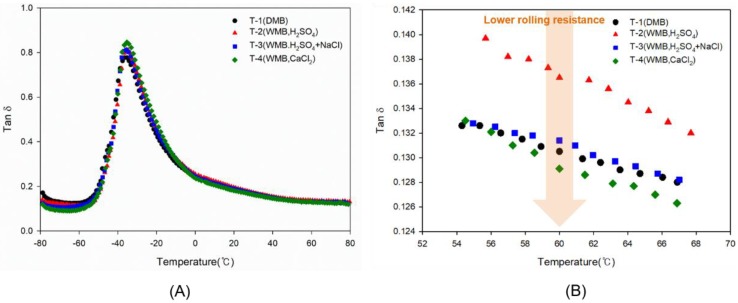
Tan δ graphs of the T-1 to T-4 compounds as a function of temperature: (**A**) from −80 to 80 °C and (**B**) from 54 to 68 °C. These graphs show that rolling resistance and wet traction performance: the black color symbol shows the hysteresis behavior of the T-1 DMB compound, the red symbol shows the hysteresis behavior of the T-2 WMB compound, the blue symbol shows the hysteresis behavior of the T-3 WMB compound, and the green symbol shows the hysteresis behavior of the T-4 WMB compound.

The results of dynamic viscoelastic properties are shown in Figure 7 and Table 12. Since only the type of coagulant was different and ESBR with the same microstructure was used in all cases, the glass transition temperature of the compound was measured to be almost the same for all samples. The value of tan δ at the glass transition temperature is higher when the dispersion of silica is better [31]. Therefore, the T-4 compound, which had the highest silica dispersion in Payne effect and Mooney viscosity measurements, showed the highest tan δ value at *T*_g_. In addition, the T-2 and T-3 compounds, both which were made using the WMB technology, showed excellent silica dispersion compared with the DMB compound, T-1. The value of tan δ at 0 °C, all compound showed similar results. 

The value of tan δ at 60 °C, which is a measure of rolling resistance during driving, generally shows a lower value as the crosslinking density is higher [39]. As a result, the DMB compound, T-1, showed the lowest value, and excellent results were obtained. The T-4 compound had a low crosslinking density compared with T-1 but had a similar rolling resistance because of the good silica dispersion and excellent F–R interaction. The results suggest that the use of calcium chloride in the WMB technology results in a lower total crosslink density than the DMB applied compound. However, as the degree of hydrophobation of the silica increases, the excellent silica dispersion and the improved filler-rubber interaction complement the low crosslinking density, showing dynamic viscoelastic properties similar to the DMB compound. 

## 4. Conclusions

The results of the analysis of silica content in ESBR/silica WMB using TGA confirmed that the silica content was 4% increased than other coagulants when alkali metal ions were present in the coagulant, leading to increased 10.1% hydrophobation of the silica by the alkali-silica reaction. 

From the results of the Payne effect measurements, it was confirmed that the ESBR/silica WMB applied compounds had a high silica dispersibility, in particular T-4 compound was improved by 25% than T-1 DMB compound when calcium chloride was used as a coagulant. Also, Mooney viscosity results showed lowest value (ML_1+4_@100 °C: 103) when calcium chloride was used, confirming the excellent silica dispersion. This occurred because the surfactant constituting the micelle in the rubber latex remained after the reaction with the coagulant, thereby acting as a processing aid and increasing the silica hydrophobation through the alkali-silica reaction. Bound rubber measurements showed more 5% bound rubber content than other compound when the WMB compound using calcium chloride due to the better hydrophobation of silica.

Measuring various properties of the vulcanizate showed that the T-2 and T-3 compounds had a loss of accelerator activity due to an acid–base reaction between residual sulfuric acid and residual emulsifier, which decreased the crosslink density. This lower crosslink density was a disadvantage compared with the WMB compound made with calcium chloride with respect to the mechanical properties, dynamic viscoelastic properties, and abrasion resistance. The T-4 compound, a WMB compound with calcium chloride, showed excellent physical properties compared with other coagulants, but the 300% modulus was 11% lower than that of the DMB compound due to the lower crosslink density because of residual calcium carboxylate. However, the high F–R interaction measured in the bound rubber test, the Payne effect, and the excellent silica dispersion measured in the Mooney viscosity test complement the properties of the WMB compound, thus exhibiting abrasion resistance and rolling resistance characteristics (tan δ at 60 °C) similar to the DMB compound.

The above results confirmed that calcium chloride was an excellent coagulant when ESBR/silica WMB was manufactured. However, the low crosslink density compared with that of the DMB compound was problematic. If this problem is solved, the WMB compound is expected to show improved the properties, dynamic viscoelastic properties, and abrasion resistance as a result of the excellent silica dispersion and F–R interaction compared with those of the DMB compound. In order to improve crosslink density, we will apply the alcohol washing process during the manufacturing process of ESBR/silica WMB to minimize residue calcium carboxylate. Also, the recovery process of waste water, organic solvent, and lost silica in the manufacturing process of ESBR/silica WMB should be studied for commercialization and optimization in plant processes [40,41].

## Figures and Tables

**Table 1:** BET surface area of TESPT modified silica according to TESPT content.

Commercial product name	Commercial source	TESPT (wt %)	BET surface area (m^2^/g)
NK136	Miraesi Inc., Gwangju, Korea	8	126
NK137		10	120
NK153		12	116
NK138		15	120

**Table 2:** Formulation of wet masterbatch (WMB) compounds and dry masterbatch (DMB) compound according to the coagulant type.

Components (phr)	DMB	WMB		
	T-1	T-2	T-3	T-4
SBR-1723	137.5	-	-	-
WMB T-2 (H_2_SO_4_)	-	219.4^(^^a)^	-	-
WMB T-3 (H_2_SO_4_ + NaCl)	-	-	220.2^(b)^	-
WMB T-4 (CaCl_2_)	-	-	-	223.0^(c)^
Pure silica	80	-	-	-
TESPT	9.6	-	-	-
ZnO	3	3	3	3
St/A	2	2	2	2
6PPD	1	1	1	1
	FMB step			
Sulfur	1.5	1.5	1.5	1.5
CBS	1.5	1.5	1.5	1.5
DPG	1.5	1.5	1.5	1.5

(a): SBR 100 phr + silica 73.1 phr + TESPT 8.8 phr + TDAE oil 37.5 phr; (b) SBR 100 phr + silica 73.8 phr + TESPT 8.9 phr + TDAE oil 37.5 phr; (c) SBR 100 phr + silica 76.3 phr + TESPT 9.2 phr + TDAE oil 37.5 phr.

**Table 3:** Mixing procedures for the DMB compound and WMB compounds.

Mixing time (min:sec)	Action of SMB step	
Compound type	DMB	WMB
0:00	Add rubber	Add WMB
0:40	Add 1/2 silica, 1/2 TESPT	-
1:40	Add 1/2 silica, 1/2 TESPT	-
3:40	Sweep	
5:40	Add ZnO, St/A, 6PPD	
12:00	Dump	
	**Action of FMB step**	
0:00	Add SMB	
0:20	Add sulfur, cure accelerators	
2:00	Dump	

**Table 4:** Degree of hydrophobation of the modified silica according to the content of TESPT.

Silica types	Without silica	Pure silica	8% TESPT	10% TESPT	12% TESPT	15% TESPT
Acid-base titration solution amount (mL)	2.22	0.95	1.25	1.68	1.85	1.91
Hydrophobation (%)	-	0	23.6	57.5	70.9	75.6

**Table 5:** pH value of WMBs according to the coagulant type.

WMB code (Coagulant)	WMB T-2 (H_2_SO_4_)	WMB T-3 (NaCl + H_2_SO_4_)	WMB T-4 (CaCl_2_)
pH	5.5	6.4	7.7

**Table 6:** Silica content in the ESBR WMB according to the type of coagulant.

WMBs	WMB T-2 (H_2_SO_4_)	WMB T-3 (NaCl + H_2_SO_4_)	WMB T-4 (CaCl_2_)
Ash (wt %)	35.1	35.7	36.4
SBR (wt %)	48.0	48.4	47.7
Silica content (phr)	73.1	73.8	76.3
Silica loss (%)	8.6	7.8	4.6

**Table 7:** Payne effect (Δ*G*′) and Mooney viscosity (ML_1+4_@100 °C) of the T-1 to T-4 compounds.

Compounds	T-1	T-2	T-3	T-4
Coagulant type	-	H_2_SO_4_	H_2_SO_4_ + NaCl	CaCl_2_
∆*G*′ (KPa) (*G*′ at 1.8% strain −*G*′ at 31.7% strain)	983.1	812.0	810.0	788.1
ML_1+4_@100 °C	114	107	104	103

**Table 8:** Bound rubber content of the T-1 to T-4 compounds.

Compounds	T-1	T-2	T-3	T-4
Bound rubber (%)	31.3	29.5	28.5	36.6

**Table 9:** Hydrophobation and calcium content according to the type of modified silica.

Silica type	Pure silica (un-modified)	12 wt % TESPT modified silica	Ca-coated 12 wt % TESPT-silica
Hydrophobation (%)	0	70.9	81.0
Ca atom content by XRF (%)	0.029	0.031	0.427

**Table 10:** Cure characteristics and crosslink density of the T-1 to T-4 compounds.

Compounds		T-1	T-2	T-3	T-4
*t*_10_	min:sec	3:32	2:17	2:10	2:02
*t*_90_	min:sec	5:35	6:27	5:38	6:02
Cure rate	N-m·min^−1^	0.656	0.325	0.512	0.690
*T*_min_	N-m	0.444	0.483	0.406	0.449
*T*_max_	N-m	1.898	1.726	1.694	1.649
*T*_max_ − *T*_min_	N-m	1.454	1.243	1.288	1.200
Crosslink density	10^−4^ mol·g^−1^	1.2178	1.1128	1.1504	1.1446

**Table 11:** Mechanical properties and DIN abrasion of the T-1 to T-4 compounds.

Compounds	Unit	T-1	T-2	T-3	T-4
*M*_100%_	kgf·cm^−2^	24.8	20.8	21.0	18.9
*M*_300%_	kgf·cm^−2^	94.1	78.5	80.3	83.4
*M*_300%_/*M*_100%_	-	3.79	3.77	3.82	4.41
Elongation at break	%	600	670	650	590
Tensile strength	kgf·cm^−2^	252	270	257	244
DIN abrasion	mg	115.1	120.5	118.2	114.0

**Table 12:** Viscoelastic properties of the T-1 to T-4 compounds.

Compounds	T-1	T-2	T-3	T-4
Coagulant type	-	H_2_SO_4_	H_2_SO_4_ + NaCl	CaCl_2_
*T*_g_ (°C)	−35.3	−35.3	−35.6	−35.6
tan δ at *T*_g_	0.7772	0.8134	0.8122	0.8461
tan δ at 0 °C	0.2452	0.2536	0.2430	0.2369
Wet traction index (%)	100	103	99	97
tan δ at 60 °C	0.1305	0.1365	0.1314	0.1291
Rolling resistance index (%)	100.0	95.6	99.3	101.8

## Supplementary Materials

The supplementary materials are available online at http://www.mdpi.com/2073-4360/10/10/1116/s1. Figure S1. TGA thermogram for the silica content in the WMBs depending on the coagulant type. In each graph, it is possible to confirm that TDAE oil, ESBR and TESPT are burned as the temperature rises, and the silica content in WMB can be estimated through the content of the remaining ash: Red line is a thermogram of WMB T-2 used H_2_SO_4_ coagulant, blue line is a thermogram of WMB T-3 used NaCl + H_2_SO_4_, and green line is a thermogram of WMB T-4 used CaCl_2_.
