# Supplementary material for: Effect of Calcium Chloride as a Coagulant on the Properties of ESBR/Silica Wet Masterbatch Compound
Source: Polymers (Basel). 2018 Oct 9;10(10):1116. doi: 10.3390/polym10101116 (PMC6404049; doi:10.3390/polym10101116)
Supplement: Supplementary file 1 [file polymers-10-01116-s001.pdf]

Supporting Information

Effects of Calcium Chloride as a Coagulant on the Properties of ESBR/silica Wet Masterbatch Compound

Woong Kim [1], Byungkyu Ahn [1], Hyunsung Mun [1], Eunho Yu [1], Kiwon Hwang [1], Donghyuk Kim [1], Gyeongchan Ryu [1], and Wonho Kim [1,*]

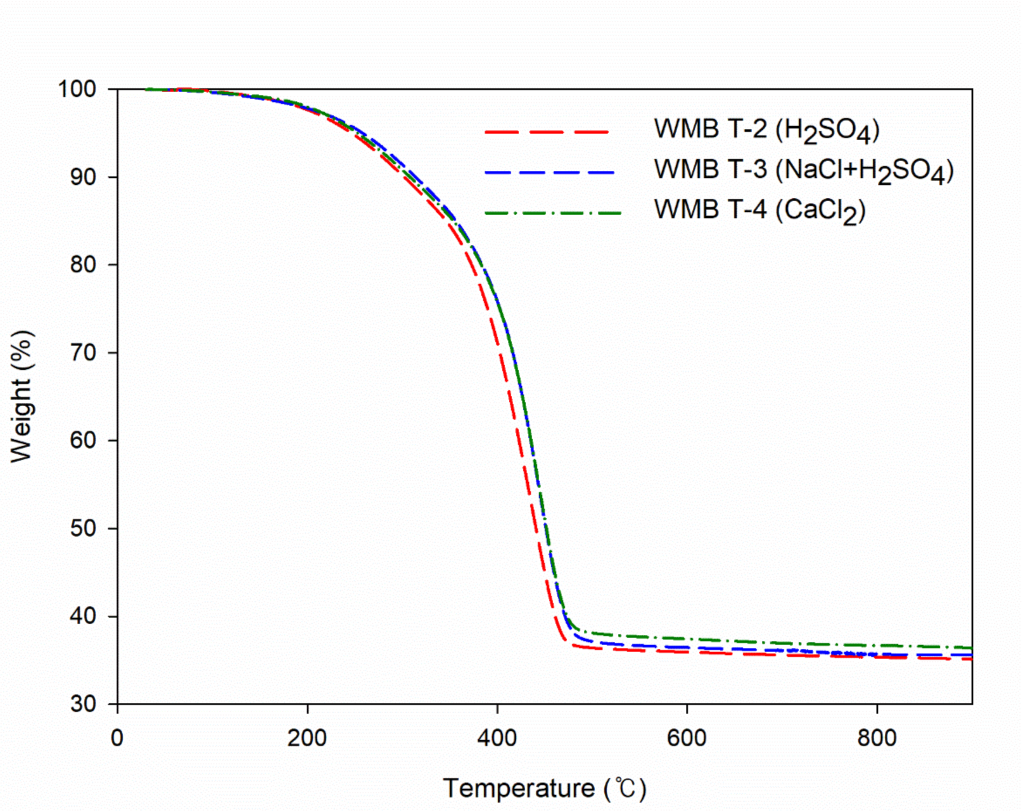

Fig. S1. TGA thermogram for the silica content in the WMBs depending on the coagulant type. In each graph, it is possible to confirm that TDAE oil, ESBR and TESPT are burned as the temperature rises, and the silica content in WMB can be estimated through the content of the remaining ash: Red line is a thermogram of WMB T-2 used H_2SO_4 coagulant, blue line is a thermogram of WMB T-3 used $NaCl+H_2SO_4$, and green line is a thermogram of WMB T-4 used $CaCl_2$.
